# Post-translational modifications of histones H3 and H4 associated with the histone methyltransferases Suv39h1 and G9a

**DOI:** 10.1186/gb-2007-8-12-r270

**Published:** 2007-12-20

**Authors:** Philippe Robin, Lauriane Fritsch, Ophélie Philipot, Fedor Svinarchuk, Slimane Ait-Si-Ali

**Affiliations:** 1 Centre National de la Recherche Scientifique (CNRS) FRE 2944, Institut André Lwoff, rue Guy Moquet, Villejuif F-94801, France; Université Paris-Sud, Villejuif F-94801, France; 2 Centre National de la Recherche Scientifique (CNRS) FRE 3018, GENETHON, bis rue de l'Internationale, Evry F-91002, France; Université d'Evry, Evry F-91002, France

## Abstract

Specific combinations of post-translational modifications of histones alter chromatin structure, facilitating gene transcription or silencing. Here we have investigated the 'histone code' associated with the histone methyltransferases Suv39h1 and G9a by combining double immunopurification and mass spectrometry. Our results confirm the previously reported histone modifications associated with Suv39h1 and G9a. Moreover, this method allowed us to demonstrate for the first time an association of acetylated histones with the repressor proteins Suv39h1 and G9a.

## Abstract 2

Mass spectrometry analysis of the post-transcriptional modifications of histones H3 and H4 that were co-purified with histone methyltransferases Suv39h1 and G9a shows that, in HeLa cells, histone methyltransferases can be physically associated with acetylated histones, which normally mark transcriptionally active chromatin.

## Background

The amino-terminal tails of nucleosomal histones protrude from the DNA and are subject to covalent modifications. These modifications include lysine acetylation, lysine and arginine methylation, serine and threonine phosphorylation, ADP-ribosylation, and ubiquitination [1]. Histone lysine methylation can have different effects depending on the residue that is modified: methylation of histone H3 at Lys4 (H3K4) is associated with gene activation, whereas methylation of H3K9, H3K27, and H4K20 generally correlates with transcriptional repression [2-4]. The roles of H3K36 and H3K79 methylation remain elusive; indeed, these modifications are associated with both transcriptional activation and repression [5,6].

Lysine residues can be mono-, di-, or trimethylated, inducing different biological responses [3,7,8]. Thus, for example, highly condensed heterochromatic regions show a high degree of trimethylated H3K9 (H3K9me3), whereas euchromatic regions are preferentially enriched in mono- and dimethylated H3K9 [2,3]. Histone lysine methylation is mediated by histone methyltransferases (HMTs), many of which contain a conserved SET [Su(var)3-9, Enhancer-of-zeste, Trithorax] domain, such as Suv39h1 (Suppressor of variegation 39h1) and G9a [1,2,9]. Suv39h1 belongs to a family of peri-centromeric proteins and is responsible for H3K9 trimethylation [10-13]. G9a (EuHMTase-2) is the major methylase responsible for mono- and dimethylation of H3K9 in euchromatic regions [14,15], but it may also be present in heterochomatic regions [16].

Covalent modifications of histones can regulate gene expression directly or through recruitment of non-histone effector proteins [2,17]. These effector proteins bind modified chromatin using a variety of chromatin-binding domains. For example, bromodomains recognize acetylated lysines, whereas chromo, MBT, Tudor, W40 domains and PHD fingers, recognize methylated lysines [17,18]. Repressive methyl-lysine modifications are recognized by chromodomain-containing proteins such as HP1 and Polycomb (PcG), which bind methylated H3K9 and H3K27, respectively, and contribute to creation of heterochromatin-like structures [19]. Thus, H3K9 methylation has been linked to both DNA methylation [20,21] and X-chromosome inactivation [22].

Different modifications of histone amino-terminal tails constitute the so-called 'histone code' [23]. Indeed, specific combinations of histone modifications can alter chromatin structure to allow transcription or to repress it, either reversibly or stably [1]. Chromatin modifications confer a unique identity on the nucleosomes involved. The composite pattern of modifications regulates the binding and activities of other chromatin-associated components. Indeed, modifications of histones at a specific nucleosome very likely influence subsequent modifications, regulated by both *cis *and *trans *mechanisms. Characterizing such modifications could provide insight into the roles of chromatin-binding proteins

In this study, we were interested in the 'histone code' associated with the HMTs Suv39h1 and G9a, as these two HMTs generally localize to two distinct regions in the nucleus. Studying modifications of the histones associated with these HMTs could help in understanding the *in vivo *state of constitutive heterochromatin associated with Suv39h1, and that of the silent euchromatin and facultative heterochromatin associated with G9a.

Our approach was to identify post-translational modifications on histones co-purified with tagged Suv39h1 and G9a HMTs. We performed a double immunopurification of these proteins from chromatin preparations enriched in mono-nucleosomes. We then studied histone modifications by a propionylation-based modification method, followed by mass spectrometry analysis [24-27].

We used four cell systems in this study: normal liver cells, HeLa cells, HeLa cells expressing a tagged form of Suv39h1, and HeLa cells expressing a tagged form of G9a. We began by comparing the global epigenetic modifications of crude nucleosomal histones isolated from these cell lines. We observed a decrease of the three repressive trimethylation marks (H3K9, H3K27 and H4K20) in cancerous HeLa cells compared with normal liver cells. HeLa cells expressing tagged Suv39h1 have a higher H3K9me3 content than the parent HeLa cells, whereas HeLa cells expressing tagged G9a show a higher level of H3K9me and non-modified H3K9. We also identified a new epigenetic modification, the monomethylation of Lys79 on histone H4. Our results help define the histone code associated with Suv39h1 and G9a. Histone H3 associated with Suv39h1 is heavily trimethylated at Lys9, whereas H3K27 and H4K20 are mainly dimethylated. In addition, Suv39h1 is associated with methylation at H3K18, H3K79 and H4K79. Histone H3 associated with G9a is mainly mono- or dimethylated at Lys9, as expected. Interestingly, we find Suv39h1 and G9a to be associated with substantial acetylation of H4K16, H3K18 and H3K23.

Taken together, our results confirm some histone modifications previously found to be associated with Suv39h1 and G9a, and show, for the first time, an unexpected association between these repressor proteins and histone acetylation, which normally activates transcription.

## Results

### Determination of global histone modifications

We first compared the basal modifications present on the crude nucleosomal histones in the different cell lines used: the cancerous HeLa cell line, and the HeLa cell lines stably expressing the H3K9-specific trimethylase Suv39h1 (HeLa-Suv39h1) or dimethylase G9a (HeLa-G9a).

**Figure 1:**
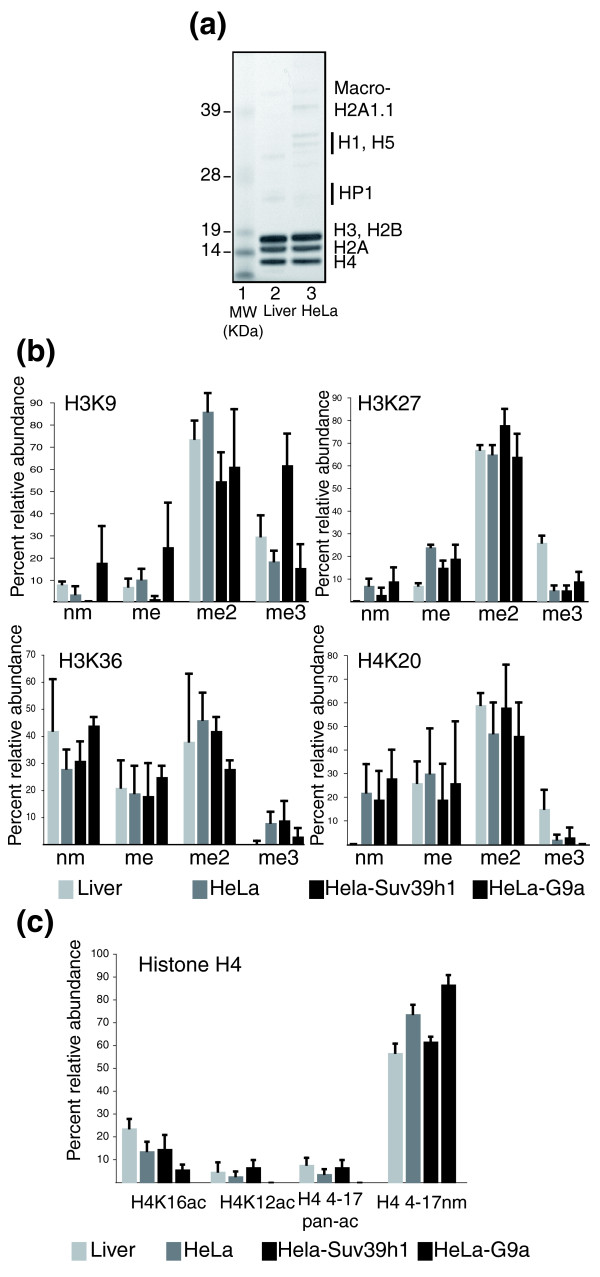
Comparison of histone H3 and H4 modifications in different cell types. **(a) **Purification of crude nucleosomal histones. Nucleosomal histones were separated on a 4-12% gradient NuPAGE gel and run in MES buffer (Invitrogen), fixed, and stained with Seeblue (Invitrogen). Lane 1, SeeBlue pre-stained molecular weight markers (Invitrogen); lane 2, nucleosomal histones from normal mouse liver; lane 3, nucleosomal histones from HeLa cells, purified on a POROS HQ column. **(b) **Methylation states of H3K9, H3K27, H3K36, and H4K20. nm, non-modified; me, monomethyl; me2, dimethyl; me3, trimethyl. Shown are the means of four independent experiments. **(c) **Basal amino-terminal modifications of histone 4 in the indicated cell types. 'H4 4-17nm': unmodified H4 peptide containing amino acids 4-17. Shown are the means of four independent experiments (± standard deviation). Pan-ac: panacetylated.

HeLa-Suv39h1 and HeLa-G9a cell lines give a different background pattern of H3K9, H3K20 and H3K27 methylation states. Indeed, our results show an approximately 40% increase in H3K9me3 in HeLa-Suv39h1 cells compared to HeLa cells (Figure 1b), whereas levels of this modification are similar in HeLa and HeLa-G9a cells (Figure 1b). In HeLa-Suv39h1 cells, H4K20me3 and H3K27me3 are present at similar levels to those found in HeLa cells (Figure 1b). When we compare HeLa-Suv39h1 to HeLa cells, the increase in H3K27me2 is similar to the decrease in H3K27me, by approximately 10-15% (Figure 1b), whereas H3K27me3 increases slightly in HeLa-G9a cells (Figure 1b). Surprisingly, in HeLa cells expressing the H3K9 dimethylase G9a, the H3K9me and non-modified H3K9 (H3K9nm) forms increase significantly, whereas H3K9me2 decreases by 21% relative to HeLa cells (Figure 1b). Generally, methylation at H3K27 and H4K20 occurs to the same extent in HeLa and in HeLa-G9a cells (Figure 1b). Methylation on H3K36 occurs at comparable levels in HeLa cells and in HeLa-Suv39h1 cells, whereas HeLa-G9a cells show a slight increase (15%) in non-modified H3K36 (Figure 1b). In HeLa-G9a cells, H3K36me2 decreases by roughly 18% compared with HeLa cells (Figure 1b).

For the amino-terminal histone H4 peptide 4-GK_5_GGK_8_GLGK_12_GGAK_16_R-17 ('peptide 4-17'), the predominant form detected in the three cell lines was a non-modified one corresponding to an ion of 1,550 m/z (Figure 1c). The most abundant single modification of this peptide is acetylated H4K16 (H4K16ac), detected as an ion of 1,536 m/z, which appears at a level of 14% in HeLa cells, 15% in HeLa-Suv39h1, and 6% in HeLa-G9a cells (Figure 1c). H4K16ac is found mostly alone, but can be found in combination with H4K8ac or with H4K12ac (data not shown). A triacetylated form of this peptide was also found, representing less than 1% of the total (data not shown). The second most abundant modification of peptide 4-17 is H4K12ac, which is found either as a single modification or in combination with H4K8ac or H4K16ac (Figure 1c, panacetyl). Considering the total monoacetylated plus panacetylated peptide, H4K12ac occurs in 5% of the peptide 4-17 in HeLa cells, 10% in Hela-Suv39h1, and less than 1% in HeLa-G9a cells. We cannot explain the much lower level observed in HeLa-G9a cells.

Finally, we found a new modification on histone H4: H4K79 monomethylation (see Additional data file 2). Indeed, approximately 20% of H4K79 is methylated in HeLa, HeLa-Suv39h1, HeLa-G9a, and normal liver cells. We confirmed this methylation by analyzing trypsin-digested histone H4 without any additional treatment. Using this method, this ion gives a poor signal, and it is detected at a level of 2% of the partially digested K79-R92 peptide. This ten-fold decrease is mostly due to the poor signal, but this ion gives a robust and complete y-series and a poor b-series in the collision fragmentation result. This modification has never been described in mammals but was suggested in *Physarum *[28]. We also detected an acetylated form of this amino acid at a level of 6% in the background cell lines.

To further validate our method, we compared the global histone modifications in normal liver cells and in the cancerous HeLa cells. This approach has been validated in previous studies of histone modifications in cancer cells [29]. Our results show a dramatic difference in the usage of the histone H3 variant H3.3, which, surprisingly, is present in 60% of the nucleosomes of normal mouse liver and in only 2-3% of nucleosomes in HeLa cell lines (data not shown). The amounts of the histone H2A variant macro-H2A seem comparable in normal liver cells and HeLa cells (Figure 1a).

We then extensively studied the three lysine methylation modifications associated with heterochromatin - H3K9me, H4K20me and H3K27me - as well as the H3K36me modification. We observed a decrease of 10-20% for the repressive trimethylation of H3K9, H3K27 and H4K20 in HeLa cells compared to normal liver cells (Figure 1b). A similar result has already been reported for H4K20 [29]. Conversely, the di- and trimethylated lysine H3K36, which are mainly associated with transcriptional activation, show an increase in HeLa cells (Figure 1b), whereas the non-modified H3K36 decreases significantly in HeLa cells compared to normal liver cells (Figure 1b).

For the amino-terminal histone H4 peptide 4-17, we also detected three different ions. The non-modified form is the predominant species in both normal liver and in HeLa cells (Figure 1c). A single acetylation at H4K16 (H4K16ac) accounts for 23% of the peptide 4-17 in liver and 14% in HeLa cells (Figure 1c). This H4K16ac modification can be found in combination with H4K8ac or with H4K12ac in another 7% of the peptide 4-17 species in mouse liver cells (data not shown). Considering the total monoacetylated plus panacetylated peptides, H4K12ac occurs 10% of the time in normal liver but only 5% in HeLa cells (Figure 1c and data not shown).

In summary, the protocol we used to study modifications of crude histone preparations, especially those of histones H3 and H4, gave satisfactory and informative results. Consequently, we used this protocol to study the histone code associated with the HMTs Suv39h1 and G9a.

### Determination of the epigenetic modifications on histones H3 and H4 associated with HMTs Suv39h1 and G9a in HeLa cells

**Figure 2:**
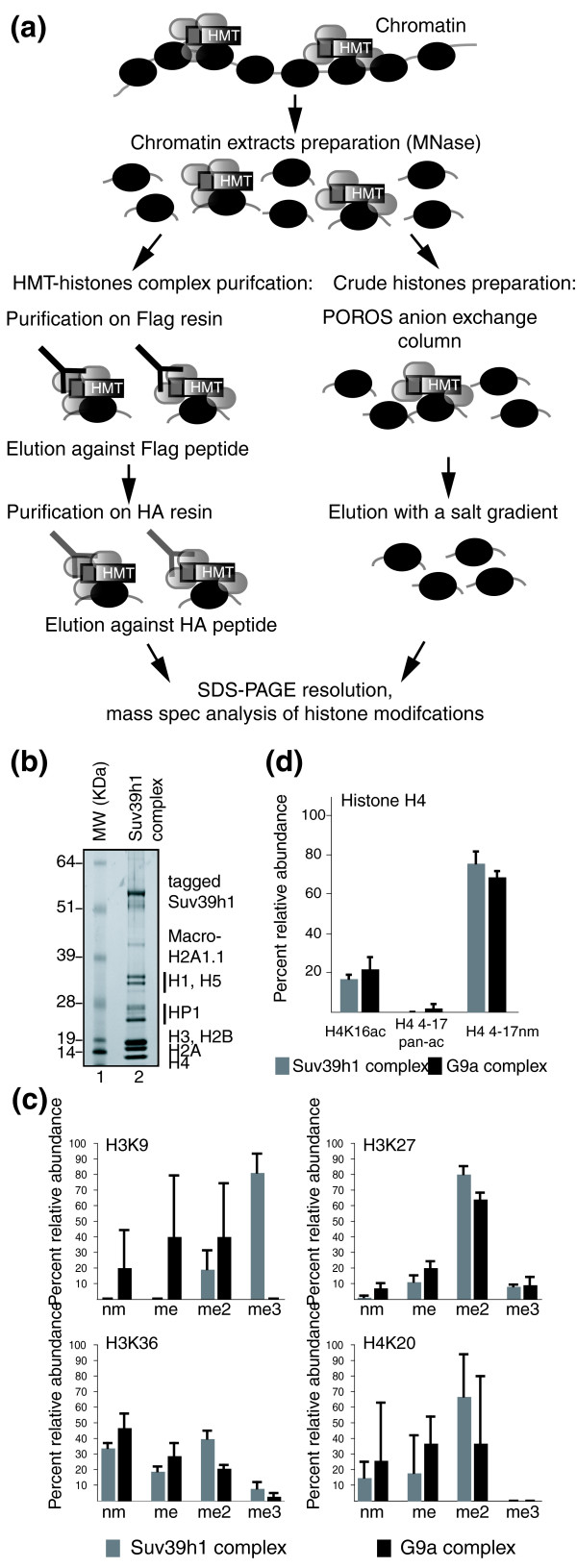
Post-translational modifications of histones H3 and H4 associated with the chromatin-binding proteins Suv39h1 and G9a. **(a) **Schematic representation of the purification protocols used to purify the HMT-histone complexes and crude histones. **(b) **Doubly immunopurified Suv39h1 complexes from chromatin extracts of 20 g of HeLa-Suv39h1 cells were resolved on a 4-12% gradient NuPAGE gel, run in MES buffer (Invitrogen), fixed, and stained with Colloidal blue. Lane 1, SeeBlue pre-stained molecular weight markers (Invitrogen); lane 2, Suv39h1 complex from chromatin fractions. **(c) **Amino-terminal lysine methylation of histones H3 and H4 associated with Suv39h1 or G9a proteins. **(d) **Post-translational modifications of histone H4 associated with Suv39h1 or G9a. Shown are the means of three independent experiments (± standard deviation).

The main goal of our study was to identify the histone modifications associated with the H3K9-specific HMTs Suv39h1 and G9a, especially on histones H3 and H4. To this end, we performed double-affinity purification of HA-Flag-Suv39h1 and HA-Flag-G9a complexes from chromatin enriched in mononucleosomes (Figure 2a and Additional data file 1). The Suv39h1-associated complex is visualized in Figure 2b, lane 2. We observe good stoichiometry of the Suv39h1-associated proteins strongly bound to chromatin, such as members of the HP1 protein family, histones H1-H5, and macro-H2A (Figure 2b). The double immunopurification has been performed on chromatin extracts from the HeLa control cell line transduced by the empty vector to measure the background signal. The results do not give any quantifiable signal, especially at the histone molecular weight (data not shown).

H3K9me3 and H4K20me2 associate with Suv39h1 at levels of 81% and 68%, respectively (Figure 2c). These percentages are approximately 40% lower in HeLa-Suv39h1 cells (compare Figures 1b and 2b, or see Additional data file 3). Position H3K27 is dimethylated (H3K27me2) 80% of the time in Suv39h1 complexes versus 65% in HeLa-Suv39h1 cells (compare Figures 1b and 2b, or see Additional data file 3). Finally, Suv39h1 is mainly associated with non-modified and dimethylated forms of H3K36 (Figure 2c).

In the protein complex associated with the HMT G9a, H3K9me and H3K9me2 both occur 40% of the time (Figure 1b), compared with 21% and 52% in HeLa-G9a cells, respectively (Figure 2c). Thus, there is a significant enrichment of H3K9nm and H3K9me in the G9a complex. We did not succeed in detecting H3K9me3 on G9a-associated histone H3, whereas this modification is detected approximately 13% of the time in HeLa-G9a cells (Figures 1b and 2b and see Additional data file 3). G9a protein is associated with the monomethylated form of H4K20, with 11% enrichment compared to the HeLa-G9a cell line. Indeed, G9a is found to be associated with H4K20me and H4K20me2 37% of the time (Figure 2c). For H3K27 and H3K36, the G9a complex gives the same distribution as its background cell line. Indeed, G9a is associated with H3K27me 20% of the time and with H3K27me2 64% of the time. At position H3K36, G9a is found with the unmodified, mono-, or dimethylated forms (Figure 2c).

In conclusion, a comparison of histone modifications associated with Suv39h1 or G9a shows that Suv39h1 is associated with H3K9me3, whereas G9a is associated with H3K9me and H3K9me2. This result is expected: Suv39h1 is a known trimethylase, and G9a a known dimethylase, of position H3K9. Surprisingly, Suv39h1 is not associated with H4K20me3.

We have also studied H3 modifications at the following positions: H3K18, which can be either acetylated or monomethylated, though we have also occasionally detected a dimethylated form (Additional data file 4); H3K23, which can only be acetylated; and H3K79, which can be monomethylated. Surprisingly, we found H3K18ac associated with both Suv39h1 and G9a complexes 9% of the time (Additional data file 5). We also detected the monomethylated form of H3K18 in Suv39h1 complexes 8% of the time, constituting an 8% enrichment, as this modification is barely detectable in HeLa-Suv39h1 cells. The monomethylated form of H3K18 has been described previously [27]. Acetylation of H3K23 is present 5% of the time in Suv39h1 and 8% in G9a complexes (Additional data file 5). Methylation of H3K79 has also been studied and was detected in association with Suv39h1 about 15% of the time, but is not detected with G9a.

Concerning histone H4, we did not find the panacetylated form of the H4 peptide 4-17 in either Suv39h1 or G9a complexes. However, we found H4K16ac associated with Suv39h1 complexes 17% of the time and with G9a complexes 22% of the time (Figure 2d). An acetyl group and a trimethyl group have comparable masses, so to confirm the H4K16ac modification, we performed a MALDI-TOF analysis on the same sample that we used for ion trapping, with internal scaling using histone peptides of non-ambiguous mass (Additional data file 6). We found the m/z ratio for the ion to be 1,536.6160. This is in agreement with the theoretical mass of an acetyl group and four propionyl groups on peptide 4-17. Thus, the signal detected on H4K16 corresponds most probably to an acetyl group.

## Discussion

Many reports to date have analyzed histone modifications by different approaches. Although these studies have improved our understanding of the role of histone modifications in biological pathways, to our knowledge few studies have sought to provide a systematic analysis of the histone modifications associated with a given chromatin-binding protein [30]. In this study, we attempted to investigate modifications of the histones H3 and H4 associated with the H3K9-specific HMTs Suv39h1 and G9a.

### Basal modifications of histones H3 and H4 in normal versus cancer cells

To validate our method, we first studied the basal histone modifications in the different cell lines used in this study. HeLa cells stably expressing tagged H3K9 tri-methylase Suv39h1 show an increase in H3K9me3 compared to HeLa cells, whereas H3K9me and H3K9me2 decrease significantly. This result is in agreement with a previous work using *suv39h*-/- cells in which the level of H3K9me3 was found to decrease, H3K9me2 was unaffected and H3K9me increased [24]. Furthermore, a study in *Drosophila *showed that a *Suv39h1 *hyperactive mutant displayed an increase in H3K9 di- and trimethylation [31]. In HeLa cells expressing tagged G9a, which is preferentially a dimethylase of H3K9, H3K9me and H3K9nm increase significantly compared to HeLa cells, whereas H3K9me2 decreases. This last result was totally unexpected, but as G9a cooperates with the other EuHMTase, GLP (EuHMTase 1), it may be necessary to co-express the two proteins to see an increase in H3K9me2. Taken together, these results suggest that Suv39h1, when over-expressed, can convert a mono- or a dimethylated H3K9 to a trimethylated state, whereas G9a can monomethylate H3K9.

H4K20me3 and H3K27me3 do not seem to change in HeLa-Suv39h1 compared to HeLa cells. And, generally, H3K27 and H4K20 methyl modifications are present to the same extent in HeLa and in HeLa-G9a cells.

We found that three of the repressive methylation modifications (H3K9me, H3K27me, and H4K20me) were underrepresented in HeLa cells and derivative lines compared to normal liver cells, whereas the activating modification H3K36me was overrepresented compared to normal liver cells. The decrease in repressive methylation is reminiscent of general DNA methylation in tumor cells [32]. Tumor suppressor gene promoters are found to be heavily methylated in tumors [33,34], and indeed there is cross-talk between H3K9 methylation and DNA methylation in many species [21,35,36]. In the case of tumor suppressor genes, it has been shown that they are also silenced by methylation on H3K9, H3K27 and H4K20 [33,37], with or without concomitant DNA methylation of the promoter. Conversely, one might think that oncogenes in tumor cells could be methylated on H3K36 and hypo-methylated on H3K9 and H3K27. It will be interesting to test whether the methylation pattern of DNA and the methylation of H3K9 and H3K27 overlap 'geographically' in tumor cells.

Finally, we report here a new modification of histone H4, the monomethylation of H4K79, which is found at a level of 20% in normal liver cells, as well as in Hela cells.

### Post-translational modifications of Suv39h1- and G9a-associated histones H3 and H4

We have studied post-translational modifications of chromatin-bound histones associated with the HMTs Suv39h1 and G9a, which overlap only partially in their nuclear distribution. Indeed, Suv39h1 is mainly located in the pericentric and constitutive heterochromatin, whereas G9a was first described as a euchromatic protein, and later was shown to have a broader distribution in the nucleus [16]. The distribution of both proteins is associated with specific methylation states of Lys9 on histone H3. When associated with Suv39h1 in constitutive heterochromatin, H3K9 is mainly trimethylated but also dimethylated; when associated with G9a in euchromatin and facultative heterochromatin, it is either non-modified, mono-, or dimethylated. We found Suv39h1 to be associated mainly with dimethylation at H4K20, but G9a was associated equally frequently with mono- or dimethylation at this position. Both Suv39h1 and G9a are associated mainly with the dimethylated form of H3K27.

Thus, Suv39h1 is mainly associated with H3K9me3, H3K27me2, and H4K20me2. These three modifications are known to act in concert to create a heterochromatin structure. At least in embryonic stem cells, Suv39h1 has been suggested to maintain H3K9 trimethylation, H3K27 monomethylation and H4K20 trimethylation at pericentromeric heterochromatin [24,38]. The apparent discrepancy between those results and ours could be explained by differences between embryonic stem cells and HeLa cells. Our working model suggests that there is a direct or indirect interaction between Suv39h1 and the HMTs responsible for H4K20 and H3K27 methylation, namely Suv4-20h and the Polycomb protein Ezh2, respectively. Indeed, a physical association between Suv39h1 and PcG proteins has been reported [39].

It has been suggested that H3K9 trimethylation constitutes the first event leading toward H4K20 trimethylation [38]. HP1 proteins, which recognize H3K9me3 created by Suv39h1, recruit Suv4-20h, the enzyme that normally establishes H4K20me3. Our results suggest that Suv39h1 is preferentially associated with H4K20me2, but not H4K20me3. This association might correspond to an intermediate state of H4K20 methylation. Another possibility is that heterochromatin modification is not homogenous; for example, some Suv39h1-bound nucleosomes may be dimethylated on H4K20, while adjacent nucleosomes are trimethylated on H4K20.

We have found a significant enrichment of H3K18ac and H3K23ac in Suv39h1-chromatin complexes. H3K23 is located within the epitope of histone H3 that is recognized by the chromodomain of Polycomb proteins [19]. Therefore, H3K23 acetylation could regulate this recognition by preventing the formation of the Polycomb complex. Indeed, distinct localizations between H3K9me3, which is associated with Suv39h1, and H3K27me3, which is recognized by Polycomb complex, have been deduced from ChIP-chip analysis [40].

In addition, we have observed an acetylated form of Lys16 of histone H4 associated with both Suv39h1 and G9a. It is quite surprising to have H4K16ac associated with the transcription repressors Suv39h1 and G9a, since this modification is mainly associated with transcriptional activation. Even so, it is unclear whether H4K16ac always causes activation, since it is associated with constitutive heterochromatin in many species [41,42], and another acetylation mark, H4K12ac, is involved in the establishment of heterochromatin in *Drosophila *[43]. Furthermore, it is known that G9a can be a coactivator [44].

It may be that acetylation at H4K16 is involved in recruiting Suv39h1 and G9a, but also other proteins, to the histone tails. For example, the chromatin remodeling complex WINAC has been described to bind H3K14ac via WSTF to induce repression of a target gene [45], and H4K16 could play a similar role in the nucleosomal context. In addition, binding of the NoRC complex to H4K16ac is required for the subsequent deacetylation of H4K5, H4K8, and H4K12 during the NoRC-dependent establishment of heterochromatin [46]. Finally, H4K16 acetylation varies in a cell cycle-dependent manner and is associated with replication [47-49]. Suv39h1 and G9a are also linked to DNA replication [50]. As H4K16 is the first lysine to be acetylated after replication [49], Suv39h1 and G9a could associate with this form in a replication-dependent manner.

We have found a new histone H4 modification: monomethylation of H4K79. H4K79me is detected in Suv39h1 and G9a complexes. This modification has never been described in mammals but was suggested in *Physarum *[28]. Mutation of H4K79 in *Saccharomyces cerevisiae *affects both telomeric and rDNA silencing [51]. In fact, H4K79 is part of the Lrs (Loss of ribosomal silencing) nucleosomal domain [52], suggesting that H4K79 methylation is associated with gene silencing. Indeed, H4K79 is located close to H3K79 in the nucleosome structure and contacts the DNA surface [51,53], suggesting that its charge is important for silencing rDNA genes [51,53]. Finally, we found H3K79me associated with Suv39h1, but not with G9a. This modification preferentially labels constitutive heterochromatin and perhaps more specifically telomeres.

## Conclusion

In conclusion, we can combine double immunopurification and mass spectrometry to uncover novel associations of histone modifications with specific chromatin-binding proteins. This method allowed us to demonstrate for the first time an association of acetylated histones with the repressor proteins Suv39h1 and G9a. It would be interesting to study the significance of such an association.

## Materials and methods

### Purification of Suv39h1 and G9a complexes

HeLa cell lines stably expressing Suv39h1 and G9a were established with human transgenes coding for full-length proteins Suv39h1 (amino acids 1-412) and G9a (amino acids 1-1,211) tagged with double-HA (haemagglutinin) and double-FLAG epitopes at the amino terminus. A HeLa control cell line transduced with the empty vector has been established and used to control the complex purification protocols. These cell lines showed about the same proliferation rate as the parent HeLa cell line.

To purify nucleosomes, we used 20 g of dry cell pellet per experiment, which corresponds roughly to 10 billion cells. Cells were resuspended in a hypotonic buffer, lysed and disrupted using 20 strokes of a tight-fitting Dounce homogenizer, and centrifuged to pellet the nuclei [54]. Suv39h1 and G9a complexes were purified as described in [55]. Briefly, nuclei were resuspended and digested with micrococcal nuclease (Sigma, Saint-Quentin Fallavier, France) until they consisted primarily of mononucleosomes (Additional data file 1). The complexes associated with nucleosomes were then purified by immunoprecipitation using anti-FLAG antibody immobilized on agarose beads (Sigma). After elution with the FLAG peptide (synthesized by Ansynth, Roosendal, The Netherlands), the bound complexes containing nucleosomes were further affinity-purified on anti-HA antibody-conjugated agarose (Sigma) and eluted with the HA peptide (synthesized by Ansynth, Roosendal, The Netherlands). The eluted protein complexes were then resolved on precast NuPAGE 4-12% bis-Tris acrylamide gradient gel in MES buffer (Invitrogen, Cergy Pontoise, France) and stained with Colloidal blue (Invitrogen, Cergy Pontoise, France). At this step, bands corresponding to histones were cut from the gel and subjected to a propionylation-based modification method (see below). The other bands were also cut from the gel, trypsin-digested using 0.4 mg of sequencing-grade trypsin (Promega, Charbonnières, France), and identified by mass spectrometry.

### Crude histone purification

Nuclei from mouse liver were prepared as described in [56]. Nuclei from liver cells and nuclei obtained from different HeLa cell lines were washed, digested with micrococcal nuclease (Sigma) at 50 units per 20 g of initial tissue or cell pellet, and sonicated for 4 minutes. Crude nucleosomes were further purified on a POROS HQ20 anion exchange column packed in a 4.6 mm × 100 mm POROS column (Applied Biosystems, Courtaboeuf, France), loaded at 0.45 M NaCl, and eluted with a salt gradient extending to 1.5 M NaCl in 50 mM Tris (pH 6.5).

### Nucleosomal histone preparation for mass spectrometry analysis

Nucleosomal histones associated with Suv39h1 or G9a, and crude histones purified from HeLa cells or from normal mouse liver, were run on a precast NuPAGE 4-12% bis-Tris acrylamide gradient gel with MES buffer (Invitrogen, Cergy Pontoise, France) and stained with Colloidal blue (Invitrogen). Gel bands corresponding to each histone were cut and destained overnight in 50% acetonitrile, 50 mM NH_4_HCO_3_. Histones were then subjected to a propionylation-based modification method [24-27]. Propionic anhydride makes covalent bonds with non-modified or monomethylated lysines and with the amino termini of proteins. Gel slices were treated for 1 h at 37°C with 100 μl of 30% propionic anhydride in methanol and 40 μl of 50 mM NH_4_HCO_3 _[24-26], followed by two ten-minute washes in 100 mM NH_4_HCO_3_, one wash in 50% acetonitrile, 100 mM NH_4_HCO_3_, and one wash in acetonitrile. The slices were then dried and digested at 37°C overnight using 0.4 μg of sequencing-grade trypsin (Promega). The digests were acidified in 0.5% trifluoroacetic acid, lyophilized, resuspended in 40 μl of 50 mM NH_4_HCO_3_, and propionylated again in 100 μl of 30% propionic anhydride in methanol for 1 h at 37°C, lyophilized and resuspended in 20 μl of 0.1% of formic acid. The second propionylation modified the newly created amino-terminal ends after trypsin digestion. These conditions gave complete lysine and amino-terminal propionylation, but also chemical methylations that can be detected using deuterated methanol (methanol-d4) for the propionic anhydride dilution (not shown).

### Determination of histone modifications by mass spectrometry

The peptide mixtures obtained as described above were run on a Nano C18 PepMap 100 pre-column (5 mm, 100 Å, 300 μm I.D. × 1 mm), coupled with a column of 75 μm I.D. × 15 cm with the same resin (LC Packings, Dionex, Voisins le Bretonneux, France). The Nano-flow-High Pressure Liquid Chromatography LC (LC Packings) is directly coupled to an electrospray ionization system on an ion-trap mass spectrometer (ESI/MS-MS; ThermoFinnigan LCQ Deca XP). The five most intense ions of the mass spectrometry scan were subjected to fragmentation (MS-MS) without any data-dependent scan. The interpretation of the mass spectrometry data was performed with the BioWorks software version 3.2 (Thermo Scientific, Courtaboeuf, France), with the following specifications: a bank of peptides from the histones cut at arginine residues was indexed with permanent add mass for the amino terminus and lysine of 56.025 Da, and three potential modifications - K minus 14.015 for acetylation or trimethylation, K+14.015 Da for a monomethylation and K minus 27.995 Da for a dimethylation. This set-up allowed us to automate analysis of the mass spectrometry raw data. Each raw dataset was analyzed to check for combinations of modifications that might have been missed by the automated method. We also took advantage of the fact that each modification shows a specific retention time on reverse phase HPLC. Ions di- and trimethylated on lysine elute before acetylated ones, propionylated ones elute later, and propionylated plus monomethylated elute last. Just one ion did not follow this rule, namely, the highly hydrophobic peptide that bears the H4K79 amino acid, for which the propionylated and methylated form elutes before the propionylated one. Retention times were used to confirm that the data analysis reconstituted the fragmentation correctly. Histone modifications were quantified by the number of ions detected by MS/MS analysis: for each post-translational modification, results are presented as the number of ions detected that bear the modification, expressed as a percentage of the total number of peptides (modified or not) recognized in the MS/MS analysis. All masses are expressed in centroid m/z values.

## Abbreviations

ac, acetylated; HA, haemagglutinin; HeLa-G9a, HeLa cells stably expressing human HA-FLAG-tagged G9a protein; HeLa-Suv39h1, HeLa cells stably expressing human HA-FLAG-tagged Suv39h1 protein; HMT, histone methytransferase; me, monomethylated; me2, dimethylated; me3, trimethylated; nm, non-modified; Suv39h1, suppressor of variegation 39h1.

## Authors' contributions

RP and AS initiated and designed this study. RP performed crude histone and complex purifications and did all the in-house mass spectrometry analysis on an ion-trap mass spectrometer. FL performed Suv39h1 and G9a complex purification. PO performed cell culture and helped in complex purification. AS established the cell lines expressing tagged proteins and set up the complex purification protocols, wrote the paper, got the supporting grants and directed the research project. SF performed the MALDI-TOF analysis to confirm the H4K16ac modification and helped in the analysis of MS data. All the authors have participated in discussing the results and reading the manuscript. All the authors have read and approved the final manuscript.

## Additional data files

The following additional data are available with the online version of this paper. Additional data file 1 shows the size of the DNA extracted from the nucleosomal preparation used to purify the Suv39h1 complex. Additional data file 2 shows the fragmentation of the ion H4K79me. Additional data file 3 shows selected amino-terminal lysine methylations of the histones H3 and H4 associated with Suv39h1 and G9a proteins compared to their background cell lines. Additional data file 4 is about the fragmentation of the 1,027 m/z ion with a propionyl group at the amino terminus, two methyl groups on lysine H3K18, and a propionyl group on H3K23. Additional data 5 shows selected histone H3 modifications in different cell backgrounds and in Suv39h1 and G9a complexes. Additional data 6 shows the H4K16ac fragmentation on a MALDI-TOF.

## Supplementary Material

Additional data file 1 The size of the DNA extracted from the nucleosomal preparation used to purify the Suv39h1 complex. Click here for file

Additional data file 2 Fragmentation of the ion H4K79me. Click here for file

Additional data file 3 Selected amino-terminal lysine methylations of the histones H3 and H4 associated with Suv39h1 and G9a proteins compared to their background cell lines. Click here for file

Additional data file 4 Fragmentation of the 1,027 m/z ion with a propionyl group at the amino terminus, two methyl groups on lysine H3K18, and a propionyl group on H3K23. Click here for file

Additional data file 5 Selected histone H3 modifications in different cell backgrounds and in Suv39h1 and G9a complexes. Click here for file

Additional data file 6 H4K16ac fragmentation on a MALDI-TOF. Click here for file
